# Advances in Emerging and Neglected Infectious Diseases

**DOI:** 10.1155/2017/1467693

**Published:** 2017-01-30

**Authors:** Charles T. Spencer, Jose Ronnie Vasconcelos

**Affiliations:** ^1^Department of Biological Sciences, University of Texas at El Paso, El Paso, TX, USA; ^2^Departamento de Biociências, Universidade Federal de São Paulo, Campus Baixada Santista, Santos, SP, Brazil

Emerging and neglected infections have proven to be highly lethal with mortality rates ranging from 10 to 80%. However, with a low incidence of disease, less public attention and support have been directed at these infections. Much more research is devoted to infections with a large public health impact, neglecting these potential diseases. Nonetheless, there are numerous infectious diseases waiting for the evolutionary or nefarious alteration that will allow them to spread readily among the human population.

At the time of conception of this special issue, the Zika virus epidemic in the Western Hemisphere has been raging for over a year continually spreading northward from Brazil and Colombia into Latin America, Mexico, and finally the United States of America. Zika has grabbed the public's attention and forced it to pay attention to this emerging disease with over 125,000 cases in 40 countries. However, the Zika virus was originally identified in 1947 with the first human cases documented in 1952 in Uganda. Subsequently, only sporadic human infections were reported until the localized Yap Island outbreak in 2007. Zika virus then receded into obscurity until the recent explosion of Zika-associated microcephaly cases. However, this is just the latest in a string of emerging or neglected diseases to bask in the stage lights of the public health community. Similarly, the Ebola virus outbreak of 2014–2016 is just the latest in a long series of local, regional, and global outbreaks spanning all the way back to 1976. We cannot ignore pathogens with low incidences of disease as these are just the pathogens that could cause the next epidemic.

Therefore, we have assembled a series of manuscripts addressing the topic of this special issue. Manuscripts published in this special issue address topics in bacterial, viral, and parasitic infections ranging from novel treatments and diagnostics to epidemiology and clinical studies.

K. C. Kosinski et al. from the United States of America present a comparative epidemiological study of several assessments indicative of a clinical diagnostic of* Schistosoma haematobium* in Ghanaian children and found agreement between a dipstick self-test and clinical diagnosis of the presence of eggs in the urine.

A. Vina-Rodriguez et al. from Germany describe a novel TaqMan-based quantitative real-time PCR assay for the diagnosis and differentiation of* Venezuelan equine encephalitis virus*. This high throughput assay could greatly facilitate detection and surveillance of VEEV.

S. Petti et al. from Italy present an epidemiological study of healthcare works tending Ebola virus victims during the last outbreak. Despite having proper training and protective equipment, some of these healthcare workers became infected without direct exposure to blood/bodily fluids. Based on their retrospective study, the authors hypothesize that these healthcare workers became infected while working with asymptomatic patients, although they cannot discount unremembered exposure to blood/bodily fluids.

E. Franceschini et al. from Italy have characterized the clinical and microbiological features of an outbreak of visceral leishmaniasis in a province in Northern Italy concluding that despite being a nonendemic area for* Leishmania infantum*, rare diseases must be considered for symptoms with unknown etiology.

D. A. Yones et al. from Egypt compared the antihelminthic activity of edible and ornamental pomegranate extracts with the goal of identifying novel and effective new candidates for the development of drugs potent against* Schistosoma mansoni*.

P. Lou et al. from China present a novel algorithm for predicting and modelling* Brucellosis *epidemics that can be used to developing possible interventions to prevent local outbreaks from developing into epidemics.

M. P. Hernández-Rivera et al. from Mexico present the results of their association study between NRAMP1 polymorphisms and cutaneous leishmaniasis. This study could lead to greater accuracy in treatment of a diverse populace with cutaneous leishmaniasis.

C. L. Hoyos et al. from Argentina present an epidemiological study of the prevalence, magnitude, and risk factors of* Leishmania/Trypanosoma cruzi* coinfection in an endemic area of Argentina.

P. Gundelly et al. from the United States of America present the clinical findings of a group of patients in the USA infected with* Rhodococcus equi* discussing changes in the prevalence and magnitude of infections.

*Charles T. Spencer* *Jose Ronnie Vasconcelos*

